# Apunipima baby basket program: a retrospective cost study

**DOI:** 10.1186/s12884-016-1133-3

**Published:** 2016-11-03

**Authors:** Kim Edmunds, Andrew Searles, Johanna Neville, Rod Ling, Janya McCalman, Jacki Mein

**Affiliations:** 1 Hunter Medical Research Institute (HMRI), Lot 1, Kookaburra Circuit, New Lambton Heights, NSW 2305 Australia; 2 Apunipima Cape York Health Council, 186 McCoombe Street, Bungalow, QLD 4870 Australia; 3 The Cairns Institute, James Cook University, PO Box 6811, Cairns, QLD 4870 Australia

**Keywords:** Maternal and child health, Indigenous, Remote, Cost study, Economic evaluation

## Abstract

**Background:**

The Baby Basket initiative was developed by Apunipima Cape York Health Council (ACYHC) to address poor maternal and child health (MCH) in Cape York, the northernmost region of Queensland. While positive outcomes for Indigenous MCH programs are reported in the literature, few studies have a strong evidence base or employ a sound methodological approach to evaluation. The aim of the cost study is to identify the resources required to deliver the Baby Basket program in the remote communities of Cape York. It represents an initial step in the economic evaluation of the Apunipima Baby Basket program. The aim of this study was to report whether the current program represents an effective use of scarce resources.

**Method:**

The cost study was conducted from the perspective of the health providers and reflects the direct resources required to deliver the Baby Basket program to 170 women across 11 communities represented by ACYHC. A flow diagram informed by interviews with ACYHC staff, administrative documents and survey feedback was used to map the program pathway and measure resource use. Monetary values, in 2013 Australian dollars, were applied to the resources used to deliver the Baby Basket program for one year.

**Results:**

The total cost of delivering the Baby Basket progam to 170 participants in Cape York was $148,642 or approximately, $874 per participant. The analysis allowed for the cost of providing the Baby Baskets to remote locations and the time for health workers to engage with women and thereby encourage a relationship with the health service. Routinely collected data showed improved engagement between expectant women and the health service during the life of the program.

**Conclusion:**

The Apunipima Baby Basket cost study identifies the resources required to deliver this program in remote communities of Cape York and provides a framework that will support prospective data collection of more specific outcome data, for future cost-effectiveness analyses and cost-benefit analyses. An investment of $874 per Baby Basket participant was associated with improved engagement with the health service, an important factor in maternal and child health.

## Background

The 2005 review of Queensland Health maternity services revealed low attendance rates of Aboriginal and Torres Strait Islander (hereafter Indigenous) women at antenatal care, higher rates of tobacco and alcohol use during pregnancy, generally poor maternal health, as well as higher rates of low birthweight and premature babies [1]. In Cape York, the northernmost region of Queensland with a large Indigenous population, maternal and child health (MCH) is particularly poor with high rates of maternal and neonatal morbidity and mortality in comparison to the rest of Australia [2].

In 2005/06, 70 % of pregnant women in Cape York were reported to have smoked at some time during their pregnancy; there were high rates of gestational diabetes and more than double the number of low birthweight babies in comparison to the rest of Queensland [3]. Regular antenatal care reduces the risk of disease or complication via early identification and treatment, as well as providing opportunities for education about healthy parenting behaviours such as good nutrition, alcohol and smoking cessation and the benefits of breast feeding. The World Health Organisation (WHO) recommends a minimum of four visits to health professionals during pregnancy [4]. Mothers who attend antenatal care regularly and early in their pregnancy are more likely to give birth to babies with normal birth weight and at normal gestation. Perinatal mortality rates also improve when women commence antenatal care earlier in their pregnancy [5]. Culturally respectful healthcare contributes to engagement with the health service and important to this engagement is relationship building, consistency of service provider, connection with the service and commitment from community elders [6].

In order to address the region’s poor maternal and child health, the Baby Basket initiative was developed by Apunipima Cape York Health Council (ACYHC). ACYHC is a regional Aboriginal community controlled health organisation (ACCHO) responsible for delivering culturally appropriate, comprehensive primary health care to 11 Cape York communities [7]. This innovative program was primarily designed to encourage Indigenous women in remote communities to present at health care centres earlier and more frequently in their pregnancy, with the aim of providing improved antenatal care as well as better mother and family education. The information provided by health workers in these interactions has the potential to assist women in achieving better maternal health and providing a better start in life for their babies [8].

The Apunipima Baby Basket program involves the provision of three baby baskets of MCH goods with associated health education resources to women in remote Cape York communities. The timing of handover corresponds with formative stages in their maternal cycle from early pregnancy to six months post-partum: 1). Antenatal at pregnancy diagnosis in the community clinic; 2). Delivery in Cairns around the time of childbirth; and 3). Postpartum during a home visit when the baby is six months old [8]. Baby basket contents are described in detail elsewhere [9]. Basket handover is a point of engagement between Indigenous women and the health service. Conducting the basket handover in local communities increases the likelihood that other members in the family will benefit from the education provided and have the opportunity to ask questions, emphasising the family centred approach. Each basket also contains a fresh food voucher valued at $40, which can only be used to purchase fresh fruit and vegetables in community stores. Each mother is entitled to a maximum of five fresh food vouchers, so the vouchers also function as an incentive for mothers to visit the clinic between baby basket handovers [2, 8].

### Economic evaluation: a cost study

While positive outcomes of Indigenous MCH programs are often reported in the literature, very few of these studies have a strong evidence base or employ a sound methodological approach to evaluation [10–15]. Such limitations can hamper economic evaluations in Indigenous MCH, making it difficult to determine the impact of specific programs on health outcomes. The need for better quality evaluations, the use of relevant indicators, and the collection of good quality longitudinal data to assess the impact of MCH programs on health outcomes for Indigenous women, infants and children are commonly raised in the literature [10, 16–20].

A retrospective evaluation of the Baby Basket program revealed improvements in key indicators in MCH [20]. While it appears that the program has achieved successful outcomes for families, the aim of the economic evaluation is to prepare an economic case for the value of the Baby Basket program, by investigating the resources required to deliver the current program. The cost study reported here represents an initial step in the economic evaluation conducted of the Apunipima Baby Basket program.

### Literature review

A brief review of the literature was conducted to inform the methods used in costing the Baby Basket program and to identify the reported costs of similar programs. CINAHL, EconLit, Informit, Medline (PubMed), Scopus, and Web of Science were the databases consulted. The following search terms were used in either the title, abstract or article: (Aborigin* or Indigen* or Torres Strait Island*) and (wellbeing or health) and (Australia) and (child or maternal or parent* or women* or pregnan* or infan*) and (program* or service*) and (economic or cost*) and (analysis or evaluation or study). In addition, websites and clearinghouses related to Indigenous maternal and child health and economic or cost analyses were searched. Finally, reference lists of articles identified by the electronic database search were hand-searched for relevant, previously unidentified sources.

The literature review highlighted the paucity of cost studies conducted of Indigenous MCH programs. Jan et al. [21] conducted an economic evaluation of the Daruk Aboriginal community midwifery service in outer western Sydney. At the time, Daruk programs included antenatal check-ups, home visits, hospital visits and delivery. The authors compared net health sector costs for Indigenous women receiving antenatal care in the Daruk midwifery service and Indigenous women receiving antenatal care at nearby services (e.g. Nepean Hospital). Patient data was gathered from medical records and the Midwives Data Collection and direct health sector costs were calculated as Daruk operational costs less the associated savings for nearby midwifery services. Downstream costs comprised use of services by Indigenous women (e.g. length of hospital stay, antenatal visits), calculated as the differences in costs incurred at Daruk service and Nepean Hospital. Cost savings were evident for the Daruk midwifery service. The study demonstrates one approach for estimating costs through comparison with other services. Such an approach relies on good co-operative relations with other services and access to patient medical records.

In more recent literature, Cannon et al. [22] developed a pregnancy simulation model to construct costs based on epidemiological pregnancy data for their obstetric population. Simulation modelling was used due to the paucity of comprehensive data (as noted above) and the small number of births in rural and remote areas. The authors compared pregnancies receiving adequate and inadequate care and their results show that the costs of programs which aim to increase access to antenatal care are likely to be cost effective. While this study was rigorous in its approach, as a simulation, the findings can only be suggestive. The authors recommend further investigation of the provision of improved antenatal care, claiming that only prospective data collection in a clinical setting could improve on their findings, something that would take considerable time to achieve.

A retrospective and prospective cohort study was conducted by Gao et al. [23] to provide data for a cost-consequences analysis which compared a baseline cohort with a more recently introduced Midwifery Group Practice (MGP). The program timeline of this cost analysis was similar to that of the Baby Basket program including the first antenatal care visit, birth, post natal care in Darwin, and in community up to six weeks after birth. The study took the perspective of the Northern Territory Department of Health and examined the direct costs of the program to compare maternity care costs pre and post introduction of the MGP. The methods employed to derive the costs were particularly rigorous, with cost assumptions (based on opinions of expert informants well versed in midwifery care in the region) used only to account for missing data [23]. The Darwin MGP was less costly, though not significantly so. However, there was an improvement in clinical outcomes, quality of care and cultural safety, maternal and child health data, Aboriginal employment, greater use of services, reduced catastrophic events and length of stay [24]. While this study utilised a pre and post study design, was more focussed on clinical outcomes, and the sample size was small, the methodological rigour employed in costing and the use of retrospective and prospective data informed the economic evaluation of the Baby Basket program.

The literature revealed very few cost studies or economic evaluations of Indigenous MCH programs, and an absence of research designed for causal inference which relates to general weaknesses in data collection and the methodology employed. Even recent economic evaluations that were otherwise methodologically rigorous suffered from a lack of data. Ideally, to minimise cost, the data used for quantitative evaluations should be based on routinely collected information and employ a suitable study design that enables meaningful comparison with a control group.

## Methods

The Baby Basket cost study was conducted from the perspective of health service providers and reflects the direct resources required to deliver the Baby Basket program to 170 women (the estimated number of baby basket recipients in 2013) across 11 communities represented by ACYHC. The cost study was based on a costing framework developed in Microsoft Excel 2010 as part of the Baby Basket evaluation (documented elsewhere) [20] that can be extended, with additional data, to support a subsequent cost-benefit analysis. This framework is part of a robust evaluation plan to accompany future iterations of the Baby Basket program, which is based on prospective routine or primary data collection and a study design, such as multiple baseline design, that better supports attributions of causality [20].

We estimated the cost of the Baby Basket program in 2013 by specifying:

- the resources considered appropriate for inclusion in the costing;
- the measurement of these resources;
- how monetary units were applied.

### Cost inclusions and exclusions

Resources included in the costing were those directly expended, compensated for or forgone by ACYHC and other health providers to deliver the Baby Basket program. Costs included materials costs for the baskets and their contents, as well as the labour required to prepare them (material and ancillary costs); the cost of the fresh food vouchers; labour costs for the midwife or Aboriginal health worker to conduct the baby basket handover; and transport costs for the baby basket to be delivered to communities. These costs represent the direct costs of the Baby Basket program, an addition to routine care costs. No additional labour costs for travel time to communities were included because baby baskets are part of ACYHC routine visits to communities. That is, the incremental difference attributable to the Baby Basket program was zero. The cost study did not explicitly include allowances for capital, depreciation or rental values for the use of community clinics where handover sometimes takes place. However, an overhead of 30 % applied to the cost of labour was assumed to cover these and other indirect costs. Due to the health system perspective of the study, the cost of women’s and other family members’ time in receiving the baby basket was excluded.

### Quantifying resources

**Fig. 1:**
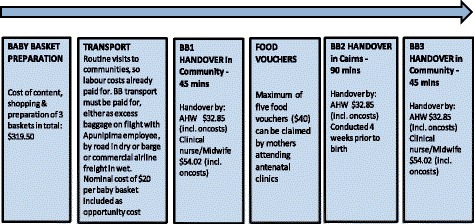
Schematic of Baby Basket Program activity pdf uploaded separately

The measurement of the quantity of resources relied on a flow diagram that mapped the program pathway, which was informed by interviews with ACYHC staff, administrative documents and survey feedback. This pathway identified six major stages of activity: i) preparation of baby baskets; ii) transportation of baby baskets; iii) - v) handover of three baby baskets; and vi) food vouchers (Fig. 1). Costing thus focussed on the resources used to prepare and deliver the three baby baskets plus the estimated value of fresh fruit vouchers provided as part of the program. The vouchers allowed the recipient to purchase fresh fruit and vegetables in community stores, up to the value of the voucher ($40). Material and ancillary costs formed an important component of the resources required to deliver the baby baskets.

Routinely available data from ACYHC facilitated cost estimates of key items. These included documentation of the materials used in each baby basket as well as the time Aboriginal health workers and other clinicians spent with mothers during engagement activities. Where data were not available, assumptions for the costing drew on information provided by experts from ACYHC, who were experienced in the delivery of MCH care in the region. The assumptions were:

- Handover of the baby basket requires at least one person, an Aboriginal health worker or other healthcare worker (ACYHC, Queensland Health, Royal Flying Doctor Service);
- Time for handover is 45 min for Baby Baskets one (BB1) and three (BB3) and 90 min for Baby Basket two (BB2) (extra time allowance to ensure preparation for impending birth and post-natal needs in Cairns);
- As Baby Basket handover involves dissemination of information that is not part of regular clinic visits, 100 % of the time for handover is attributed to the program;
- Health worker travel time for handover is not included in the costing. Aboriginal health workers are located at community clinics, or in nearby communities, so their travel time to each handover is negligible. While some baskets were handed over by a nurse or midwife who, in most cases, travel from Cairns, this additional distance was not included in the costing. This travel was assumed to be part of routine clinic visits, which occur regardless of whether there is a baby basket to deliver;
- Transport costs for baby baskets were costed to the program. In practice, these varied according to a number of factors such as baby basket number (BB1, BB2 or BB3), location of community and season - some communities are accessible only by barge during the wet season. In recognition of the cost of basket transport, a nominal cost of $20 per basket was applied. This amount was derived in consultation with ACYHC staff.
- If a woman receives BB1, she will also receive BB2 and BB3. Qualitative evaluations of the program show that women appreciate the baby baskets, which were an added incentive to attend the clinic [2, 8, 20]. Surveys conducted by ACYHC during face-to-face interactions between healthcare workers and women receiving baby baskets between 2009 and 2013 provided data on the number of baskets.
- On average, women attend two clinics to receive food vouchers, each valued at $40, in addition to the three included in the baby baskets. That is, all women receive the maximum five vouchers, however, only 70 % were used. The estimate of 70 % utilisation was based on ACYHC records from 2012.

### Monetary units

Monetary values, in 2013 Australian dollars, were applied to the resources used to deliver the Baby Basket program. We used opportunity cost, which is the value of activity foregone because of the resources committed to the program. Market price is an appropriate proxy for the opportunity cost of resources [25]. Market prices for labour were based on current wage and salary scales for nurses, midwives, and Aboriginal health workers.

### Modelling

All modelling was undertaken in Microsoft Excel 2010. Costs were estimated for one year in which 510 baby baskets were delivered to 170 participants. The modelling was based on a bottom-up approach whereby unit costs were summed to arrive at the total cost. Average cost was calculated by dividing total cost by the number of baby baskets delivered in 2013 (Table 1).

**Table 1:** Estimated cost of Baby Basket program for 170 participants

Program stage	Item	Value	Average annual cost
Baby Basket 1 (BB1)	Materials and ancillary costs per BB1	$194	
	Total materials and ancillary costs BB1	$33,040	
	Total labour costs BB1	$5373	
	Total cost BB1 (average year)		$38,413
Baby Basket 2 (BB2)	Materials and ancillary costs per BB2	$220	
	Total materials and ancillary costs BB2	$37,363	
	Total labour costs BB2	$9773	
	Total cost BB2 (average year)		$47,136
Baby Basket 3 (BB3)	Materials and ancillary costs per BB3	$197	
	Total materials and ancillary costs BB3	$33,414	
	Total labour costs BB3	$5880	
	Total cost BB3 (average year)		$39,234
Program costs (combined BB1 BB2 BB3)	Total materials and ancillary costs	$103,816	
	Total labour cost	$21,026	
	Total Baby Basket costs		$124,842
Food Vouchers	Food voucher cost	$40	
	5 food vouchers for 170 participants	$34,000	
	Total food voucher costs (70 % use)		$23,800
BALANCE	Annual program costs (170 participants)		$148,642
	Per participant cost (based on 170 participants; 3 baskets; food vouchers)		$874

## Results and discussion

The total cost of delivering the Apunipima Baby Basket progam to 170 participants in remote communities in Cape York was $148,642 or approximately $874 per participant. It was assumed that each participant received all three baby baskets and five food vouchers, 70 % of which were utilised. Materials and ancillary expenses formed the largest single component of costs. Labour costs varied, with the second baby basket (BB2) requiring a greater input of labour (Table 1). The analysis allowed for the cost of providing the baby baskets to remote locations and the time for health workers to engage with women and thereby encourage a relationship with the health service.

As the literature review revealed, there is little available data and few cost studies of Aboriginal and Torres Strait Islander MCH programs from which to draw comparison with the Apunipima Baby Basket cost study. Even recent cost analyses that were methodologically rigorous suffered from a lack of data. Poor data undermines the ability to undertake a systematic and rigorous evaluation. Ideally, economic evaluation is based on prospective data, routinely collected, and employs a study design that incorporates a control group for meaningful comparison, permitting some attribution of causality.

This cost analysis has a number of limitations. Much of the information used to model the cost of providing the Baby Basket program was estimated using the best available information. While time, combined with the number of remote locations participating in the Baby Basket program, did not allow for prospective data collection, the availability of routinely collected data contributed to robust retrospective cost estimates. Where data were not available, assumptions were informed by MCH experts from ACYHC experienced in the delivery of maternal and child health care in the region. In the future, cost accounting techniques for accurate expense recording for filling and delivering the baby baskets would improve accuracy. The current costing framework will also support updating based on prospective data collection. An evaluation framework (discussed elsewhere) [20] has been developed for future iterations of the Baby Basket program that will address the limitations of the current cost study and contribute to a more rigorous economic evaluation in the future.

## Conclusion

In order to address the region’s poor maternal and child health, the Baby Basket initiative was developed by ACYHC. This innovative program was primarily designed to encourage Indigenous women in remote communities to present at health care centres earlier and more frequently in their pregnancy, with the aim of providing improved antenatal care as well as better mother and family education. This cost study identified the resources required to deliver the Baby Basket program in the remote communities of Cape York, Queensland. It uncovers policy relevant information by identifying the resources required to transfer the Baby Basket program to other remote locations. Importantly, the Baby Basket cost study provides a framework that will support updating based on prospective data collection. In addition, with the collection of more specific outcome data, the framework will provide a basis for conducting future cost-effectiveness analyses and cost-benefit analyses. In light of other published research on the effectiveness of the Apunipima Baby Basket program based on selected qualitative and quantitative outcomes [20], the evidence suggests that value for money has been achieved as more women are engaging with the health service. For an investment of $874 per Baby Basket participant, an opportunity has been created to improve maternal health and provide infants with a better start in life.

## Abbreviations

ACYHC: Apunipima Cape York Health Council
BB1: Baby basket one
BB2: Baby basket two
BB3: Baby basket three
MCH: Maternal and child health
